# Ventricular Fibrillation From Spontaneous Coronary Artery Dissection in a Patient With Atrioventricular Nodal Re-entrant Tachycardia: Rationale for Implantable Cardioverter Defibrillator Therapy

**DOI:** 10.7759/cureus.98081

**Published:** 2025-11-29

**Authors:** Rehan Akhtar, Jennifer Llewellyn, Matthew Grant

**Affiliations:** 1 Department of Medical Education, Royal Brompton Hospital, London, GBR; 2 Department of Cardiology, Liverpool Heart and Chest Hospital, Liverpool, GBR; 3 Department of Cardiology, Countess of Chester Hospital, Chester, GBR

**Keywords:** atrioventricular nodal re-entrant tachycardia, implantable-cardioverter defibrillator, multimodality cardiac imaging, spontaneous – coronary artery – dissection – etiology – acute coronary syndrome, ventricular fibrillation arrest

## Abstract

Ventricular fibrillation (VF) management requires identification of the underlying cause and consideration of implantable cardioverter defibrillator (ICD) therapy; these decisions are complicated when the aetiology is unclear. We present a 41-year-old woman with lifelong atrioventricular nodal re-entrant tachycardia (AVNRT) who experienced cardiac arrest secondary to VF. Multimodality cardiac imaging identified features suggestive of spontaneous coronary artery dissection (SCAD) with transient vessel healing but a new apical scar. Given the scar-related arrhythmogenic substrate, risk of SCAD recurrence, and prior AVNRT episodes degenerating into polymorphic ventricular tachycardia (PMVT), a single-chamber ICD was implanted. This case highlights the need to avoid diagnostic anchoring on the pre-existing arrhythmia as the cause for cardiac arrest and demonstrates the value of imaging in identifying SCAD as the cause and in guiding ICD decision-making.

## Introduction

Discovering the cause of ventricular fibrillation (VF) is important for long-term management. Current guidelines support implantable cardioverter defibrillator (ICD) insertion for patients with no reversible cause who are at high risk of recurrence to prevent sudden cardiac death [1]. The decision becomes challenging when the cause is unclear. We report a case of VF secondary to spontaneous coronary artery dissection (SCAD) where the diagnosis was confounded by a history of supraventricular arrhythmia. We discuss the role of imaging and the rationale for an ICD. 

## Case presentation

A 41-year-old woman presented to the emergency department in cardiac arrest. The initial rhythm was VF, and return of spontaneous circulation was achieved following defibrillation. A collateral history reported presyncope and chest pain for several hours prior to the collapse, triggered by an argument with a family member. 

**Figure 1:**
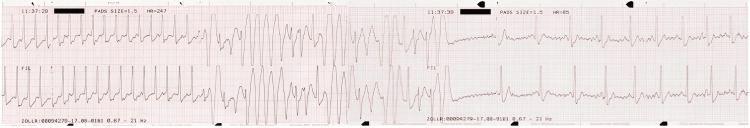
Rapid atrioventricular nodal re-entrant tachycardia at 247 beats per minute, followed by a premature ventricular complex precipitating degeneration into polymorphic ventricular tachycardia

There was a background of atrioventricular nodal re-entrant tachycardia (AVNRT) since the age of nine. There was no family history to suggest a genetic cause, and no risk factors for coronary artery disease (CAD). Her baseline ECGs had never demonstrated pre-excitation, and the QT interval was normal. She had presented three years prior with symptomatic AVNRT, which degenerated into polymorphic ventricular tachycardia (PMVT) during Valsalva manoeuvre before terminating into sinus tachycardia (Figure 1). She had declined electrophysiological (EP) studies at the time and was discharged on a low-dose beta blocker. 

**Figure 2:**
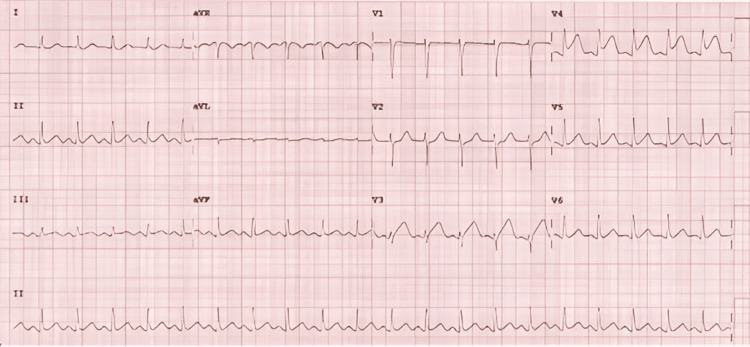
Initial post-cardiac arrest electrocardiogram showing high take-off ST segments in V3 and V4

**Figure 3:**
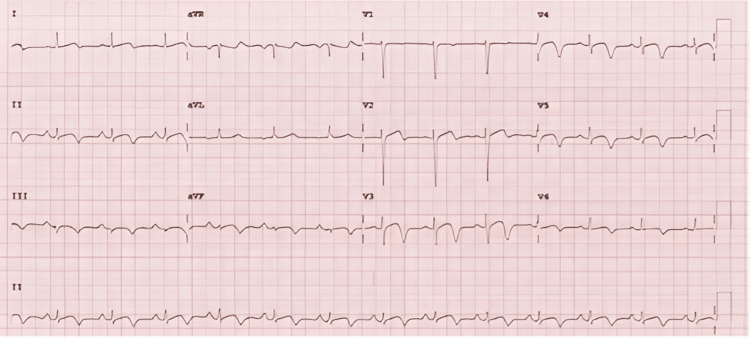
Subsequent ECG six hours later with deep anterolateral and inferior T-wave inversion, raising further suspicion of myocardial infarction as the aetiology of the cardiac arrest

The electrocardiogram (ECG) immediately post-cardiac arrest showed high take-off anterior ST segments with no significant elevation (Figure 2). The ECG six hours later evolved with T-wave inversion in the anterolateral and inferior leads (Figure 3). The initial troponin I was 10,000 ng/L (normal <13 ng/L), which, in combination with the ECGs, raised suspicion for underlying ischaemia or takotsubo cardiomyopathy, particularly as this was following an emotional trigger. A transthoracic echocardiogram took place 24 hours later, which demonstrated an akinetic apex with otherwise normal left ventricular function.

**Figure 4:**
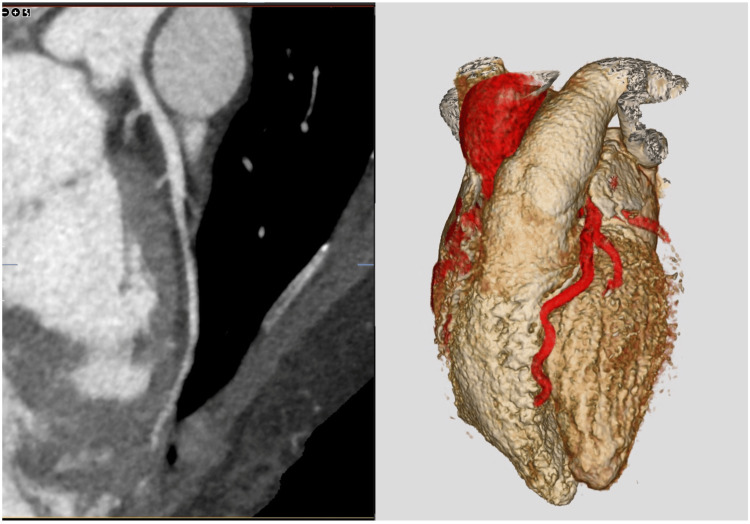
Computed tomography coronary angiography images demonstrating early termination of left anterior descending artery in the interventricular groove, suggesting spontaneous coronary artery dissection

She underwent a CT coronary angiogram (CTCA) 48 hours after presentation. This demonstrated abrupt termination of the left anterior descending (LAD) artery in the mid-distal segment, consistent with SCAD. There was no evidence of underlying atheromatous CAD on this imaging (Figure 4). 

**Figure 5:**
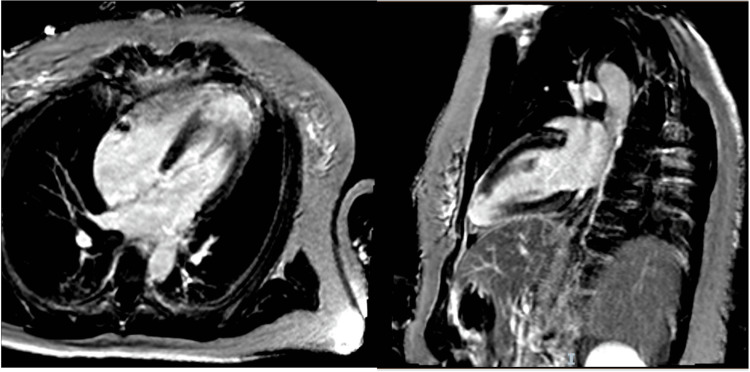
Cardiac magnetic resonance imaging confirming evidence of an apical infarction in phase-sensitive inversion-recovery sequence in apical four-chamber and two-chamber views

Two weeks later, the patient was transferred to a tertiary cardiac centre for further work-up and management. She underwent cardiac MRI here, which confirmed acute myocardial infarction in the distal LAD territory with active myocardial oedema and apical akinesia (Figure 5). Left ventricular function was preserved. A repeat CTCA demonstrated normal coronary arteries with no evidence of dissection and a recanalised LAD. A bubble echocardiogram did not demonstrate an intra-cardiac shunt. A paroxysmal atrial arrhythmia was not identified on continuous electrocardiogram monitoring over a three-week period. Following multidisciplinary team discussion, the patient had a single-chamber transvenous ICD implanted. 

## Discussion

The cardiac arrest was initially considered secondary to longstanding AVNRT triggering PMVT, a rhythm known to frequently progress to VF [2]. This phenomenon in the absence of structural heart disease is rare and has only been documented in a few case reports [3-4]. Several mechanisms have been proposed, including high sympathetic tone, rate-related shortening of the ventricular refractory period and relative ischaemia during rapid AVNRT, resulting in myocardial irritability [4]. The first ECG changes were thought to represent myocardial stunning post-cardiac arrest and resuscitation. However, there was increasing evidence of myocardial infarction with an evolving ECG several hours later and a significantly elevated troponin, out of proportion for cardiac arrest alone. The patient was treated with anti-platelet agents pending further urgent coronary artery assessment. 

There were no risk factors for atherosclerotic plaque rupture in our patient, so this was felt to be an unlikely cause of infarction. The leading differential diagnoses were therefore SCAD, a paroxysmal atrial arrhythmia which had resulted in coronary artery embolism or takotsubo cardiomyopathy. CTCA and transthoracic echocardiography were chosen as the initial investigations as they were readily available in our centre and are non-invasive. 

There were no overt features of takotsubo cardiomyopathy on initial echocardiography, and left ventricular function was normal; this was later supported and ruled out on the cardiac MRI. Prolonged ECG monitoring also made atrial arrhythmia resulting in an embolism unlikely, and there were no filling defects to support an embolism on subsequent CTCA. The probable diagnosis was consequently SCAD, which was later supported by CTCA. SCAD is caused by a tear in the coronary artery wall, forming a false lumen; it is typically triggered by an external stressor and often resolves spontaneously [5]. There is increasing evidence that invasive angiography with intracoronary imaging (intravascular ultrasound or optical coherence tomography) can confirm the presence of SCAD and determine the location of an intimal flap with better spatial resolution than CT [5]. There is a theoretical risk of further SCAD propagation with invasive angiography, so CTCA may be preferential when assessing for resolution [6]. 

The use of ICDs in patients with underlying SCAD is controversial, and a conservative approach is recommended for most patients [7]. Of the 3,978 patients with SCAD included in a recent meta-analysis, 35 had an ICD, and there was only one appropriate defibrillation recorded on prolonged follow-up [8]. They also report that the risk of recurrent myocardial infarction and recurrent SCAD is higher in those who present in cardiac arrest than in those who do not [8]. A multidisciplinary team discussion concluded that our patient was at high risk of VF recurrence due to her apical scar acting as an arrhythmia substrate, the risk of SCAD recurring and the small risk of her AVNRT degenerating into PMVT (despite high-dose beta blockade and pending further EP studies). 

ICDs are typically implanted transvenously via the subclavian vein, with a lead positioned in the right ventricle [1]. Mechanical stress on the lead contributes to a failure rate of up to 40% within five years, with the lead also implicated in infection risk and tricuspid valve dysfunction [9]. Subcutaneous ICDs eliminate the risks associated with transvenous leads by using an external substernal lead and are often preferred in younger patients because of this [10]. However, these devices have a similar longevity of 5-7 years and are significantly more costly in Europe compared to transvenous ICDs [9]. Inappropriate shocks remain a potential complication for both systems. Subcutaneous devices require pre-screening to ensure they can accurately discern the QRS complex from the T-wave, reducing the risk of inappropriate shock delivery from over-sensing the T-wave [9]. Our patient failed screening in two of the three vectors, so a transvenous ICD was inserted. 

## Conclusions

This case highlights key clinical lessons in the evaluation of cardiac arrest when potential arrhythmogenic and ischaemic mechanisms coexist. It demonstrates the need to avoid diagnostic anchoring on the prior arrhythmia and to pursue objective reassessment when imaging or biomarkers suggest an alternative cause. Multimodality imaging is critical when the aetiology of cardiac arrest is uncertain; computed tomography, coronary angiography and cardiac magnetic resonance imaging can distinguish SCAD from an atherosclerotic plaque rupture event or takotsubo cardiomyopathy. Finally, ICD decisions in SCAD are individualised, although conservative management is typical, an ICD is reasonable when there are additional risk factors that increase the likelihood of malignant ventricular arrhythmias.

## References

[1] Zeppenfeld K, Tfelt-Hansen J, de Riva M (2022). 2022 ESC Guidelines for the management of patients with ventricular arrhythmias and the prevention of sudden cardiac death. Eur Heart J.

[2] Viskin S, Chorin E, Viskin D, Hochstadt A, Schwartz AL, Rosso R (2021). Polymorphic ventricular tachycardia: Terminology, mechanism, diagnosis, and emergency therapy. Circulation.

[3] Oomen AW, van Huysduynen BH, Sy RW (2019). Supraventricular tachycardia triggering polymorphic ventricular tachycardia. HeartRhythm Case Rep.

[4] Moondra V, Sangha R, Greenberg ML (2011). Spontaneous deterioration of atrioventricular nodal reentrant tachycardia to polymorphic ventricular tachycardia in the absence of heart disease. Pacing Clin Electrophysiol.

[5] Offen S, Yang C, Saw J (2024). Spontaneous coronary artery dissection (SCAD): A contemporary review. Clin Cardiol.

[6] Al Emam AR, Almomani A, Gilani SA, Khalife WI (2016). Spontaneous coronary artery dissection: one Disease, variable presentations, and different management approaches. Int J Angiol.

[7] Garg J, Shah K, Shah S, Turagam MK, Natale A, Lakkireddy D (2021). Implantable cardioverter-defibrillator in patients with spontaneous coronary artery dissection presenting with sudden cardiac arrest. J Cardiovasc Electrophysiol.

[8] Baqal O, Karikalan SA, Hasabo EA (2025). In-hospital and long-term outcomes in spontaneous coronary artery dissection with concurrent cardiac arrest: Systematic review and meta-analysis. Heart Rhythm O2.

[9] McLeod CJ, Boersma L, Okamura H, Friedman PA (2017). The subcutaneous implantable cardioverter defibrillator: state-of-the-art review. Eur Heart J.

[10] van Dijk VF, Boersma LV (2020). The subcutaneous implantable cardioverter defibrillator in 2019 and beyond. Trends Cardiovasc Med.

